# Patient characteristics and acute cardiovascular event rates among patients with very high‐risk and non‐very high‐risk atherosclerotic cardiovascular disease

**DOI:** 10.1002/clc.23706

**Published:** 2021-08-05

**Authors:** Gregg C. Fonarow, Mikhail N. Kosiborod, Pallavi B. Rane, Sasikiran Nunna, Guillermo Villa, Mohdhar Habib, Jorge Arellano, Katherine E. Mues, Kainan Sun, Rolin L. Wade

**Affiliations:** ^1^ Ahmanson‐UCLA Cardiomyopathy Center, David Geffen School of Medicine University of California Los Angeles California USA; ^2^ Saint Luke's Mid America Heart Institute and University of Missouri‐Kansas City Kansas City Missouri USA; ^3^ Amgen Inc. Thousand Oaks California USA; ^4^ IQVIA Plymouth Meeting Pennsylvania USA; ^5^ Amgen (Europe) GmbH Zug Switzerland

**Keywords:** atherosclerotic cardiovascular disease, major cardiovascular events, real‐world evidence, very high‐risk

## Abstract

**Background:**

The risk for subsequent major cardiovascular (CV) events among patients with very high‐risk (VHR) atherosclerotic CV disease (ASCVD) remains to be fully elucidated.

**Hypothesis:**

We assessed the characteristics and major CV event rates of patients with VHR versus non‐VHR ASCVD in a real‐world setting in the United States (US), hypothesizing that patients with VHR ASCVD would have higher CV event rates.

**Methods:**

This was a retrospective cohort study conducted from January 01, 2011, to June 30, 2018, in the US using the Prognos LDL‐C database linked to the IQVIA PharMetrics Plus® database supplemented with the IQVIA prescription claims (Dx/LRx) databases. Patients were ≥18 years old and had  ≥2 non‐ancillary medical claims in the linked databases at least 30 days apart. The study was conducted in 2 stages: (1) identification of patients with ASCVD who met the definition of VHR ASCVD and a matched cohort of non‐VHR ASCVD patients using the incidence density sampling (IDS) approach; (2) estimation of the occurrence of major CV events.

**Results:**

Among patients with ≥1 major ASCVD event (*N*=147,679), most qualified as VHR ASCVD (79.5%). There were 115,460 patients each in IDS‐matched VHR and non‐VHR ASCVD cohorts. The composite myocardial infarction/ischemic stroke event rates in the VHR and non‐VHR ASCVD cohorts were 8.04 (95% confidence interval [95% CI]: 7.87‐8.22) and 0.82 (95% CI: 0.77‐0.88) events per 100 patient‐years, respectively, during the 1‐year post‐index period.

**Conclusions:**

Most patients with ≥1 previous major ASCVD event treated in real‐world US clinical practice qualified as VHR ASCVD. Patients with VHR ASCVD had much higher rates of major CV events versus non‐VHR ASCVD patients.

## 1 INTRODUCTION

Low‐density lipoprotein cholesterol (LDL‐C) is a modifiable causal risk factor in the pathogenesis of atherosclerotic cardiovascular (CV) disease (ASCVD),^1^ with lower LDL‐C levels associated with a reduced risk of CV events and improved patient outcomes.^2^, ^3^, ^4^, ^5^, ^6^ Updates in the 2018 American College of Cardiology/American Heart Association (ACC/AHA) multi‐society blood cholesterol guideline introduced the very high‐risk (VHR) ASCVD category.^7^ These patients have a history of multiple major ASCVD events (i.e., recent acute coronary syndrome [ACS] ≤12 months, history of myocardial infarction [MI] >12 months, history of ischemic stroke [IS], or symptomatic peripheral arterial disease [PAD]), or a single major ASCVD event and multiple high‐risk conditions.^7^ The ACC/AHA 2018 guideline recommends that all patients with VHR ASCVD receive lipid‐lowering therapy (LLT) with high‐intensity or maximally tolerated statin therapy.^7^ For patients with VHR ASCVD with LDL‐C ≥70 mg/dL (≥1.8 mmol/L) despite optimized statin therapy, the addition of ezetimibe and proprotein convertase subtilisin/kexin type 9 (PCSK9) inhibitors is recommended.^7^

Considering the recent introduction of the VHR stratification,^7^ the clinical characteristics, including treatment patterns and risk for subsequent major CV events, among patients with VHR versus those with non‐VHR ASCVD remain to be fully elucidated in routine clinical practice. Real‐world characterization of the VHR ASCVD population is important as these patients are likely to benefit from intensive LLT with the addition of non‐statin therapies such as PCSK9 inhibitors.^4^, ^5^, ^6^, ^8^, ^9^ Therefore, the current study had two objectives: first, to describe patient characteristics, utilization of LLT, and LDL‐C levels among patients with ASCVD who met the definition of VHR per the 2018 ACC/AHA cholesterol guideline^7^ versus patients with ASCVD not meeting the VHR criteria; and second, to estimate the rates of subsequent major CV events in VHR ASCVD and non‐VHR ASCVD cohorts, with analyses by type of major ASCVD event.

## 2 METHODS

### 2.1 Study design and patients

This retrospective cohort study was conducted in the United States (US) using the Prognos LDL‐C database (Prognos Health, New York, NY, USA)^10^ linked to the IQVIA PharMetrics® Plus database supplemented with the IQVIA prescription claims (Dx/LRx) databases (IQVIA, Plymouth Meeting, PA, USA).^11^ The Prognos LDL‐C database has been previously used in retrospective cohort studies.^12^, ^13^, ^14^ The aggregated IQVIA PharMetrics Plus database comprises adjudicated claims for patients across the US and is sourced directly from insurance companies, and contains data on patient's health plan claims, demographics, clinical characteristics, and occurrence of CV events. The IQVIA LRx database captures information on adjudicated dispensed prescriptions sourced from retail, mail, long‐term care, and specialty pharmacies. The IQVIA Dx database contains unadjudicated medical claims from office‐based physicians, ambulatory facilities, and hospital‐based physicians, and is sourced from clearing houses involved in the claims processing. The IQVIA databases linked to the Prognos LDL‐C database were accessible to the authors of this study, and a primary study dataset was constructed using linked patient data from the databases.

The study included patients aged ≥18 years with a measured LDL‐C level between January 01, 2016, to June 30, 2018, along with ≥2 non‐ancillary medical claims from the IQVIA PharMetrics Plus database at least 30 days apart during the overall study period (January 01, 2011, to June 30, 2018). Eligible patients were required to have a diagnosis of ASCVD (identified using ≥1 inpatient [IP]/outpatient [OP] medical claims with an International Classification of Diseases [ICD]‐9, ICD‐10, and/or Current Procedural Terminology [CPT] diagnosis code for ASCVD [Supplementary Table 1]) between January 01, 2011, and their most recent LDL‐C test date.

**FIGURE 1:**
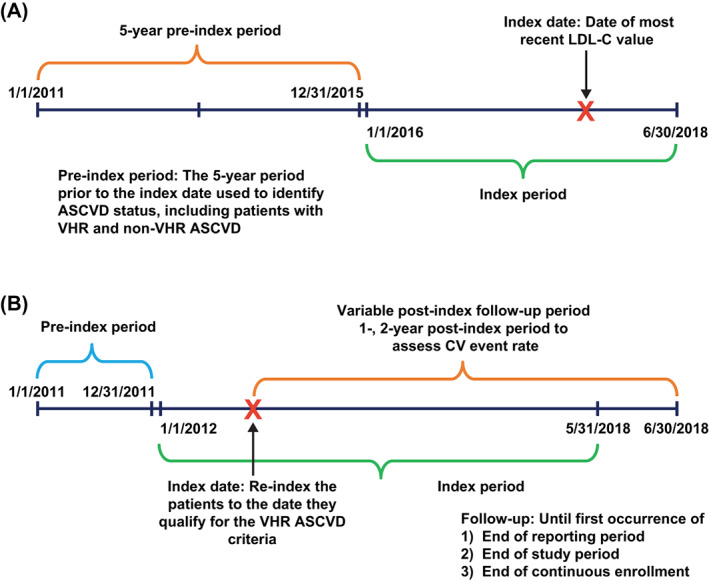
Study design for (A) stage 1: identification of VHR and non‐VHR ASCVD patients; (B) stage 2: estimation of CV event rates. ASCVD, atherosclerotic cardiovascular disease; CV, cardiovascular; LDL‐C, low‐density lipoprotein cholesterol; VHR, very high‐risk

The study was conducted in two stages (Figure 1). In stage 1 of the study, patients with VHR ASCVD were identified by the presence of the 2018 ACC/AHA guideline criteria^7^ for VHR ASCVD (i.e., 1 major ASCVD event and 2 risk factors, or ≥2 major ASCVD events) during the 5‐year pre‐index period (January 01, 2011, to December 31, 2015). The operational definitions used to identify the VHR ASCVD criteria are reported in Supplementary Table 2. The index date was the date of the most recent LDL‐C value during the index period (January 01, 2016, to June 30, 2018) (Figure 1).

To obtain comparable cohorts, patients with VHR ASCVD were matched to a cohort of non‐VHR ASCVD patients with comparable patient demographics (age, sex, region) in a 1:1 ratio using the incidence density sampling (IDS) method, also referred to as the risk set sampling method.^15^, ^16^, ^17^ The IDS methodology followed a “sampling with replacement approach,” where a patient with non‐VHR ASCVD could be matched to multiple patients with VHR ASCVD, and patients sampled in the non‐VHR cohort were eligible to become patients with VHR ASCVD at a later date. The rationale for using the IDS methodology was to obtain matched controls with similar risk as cases, allowing for unbiased estimates of CV events. Specific advantages of the IDS method included the reduction of selection bias, where other methods such as cumulative density sampling could have resulted in patients in the non‐VHR ASCVD cohort having a low likelihood of CV events, as patients who met the VHR ASCVD criteria at any point during the study period would not have been included in the risk set of patients for non‐VHR cohort selection. The ability of patients with non‐VHR ASCVD to become a VHR ASCVD patient at a later date resulted in a cohort of comparable risk and provided an unbiased estimate of CV events in patients with non‐VHR ASCVD.

To match a non‐VHR ASCVD patient with a VHR ASCVD patient, we first identified all patients in the study cohort who were at risk on the index date (diagnosed with ASCVD, not lost to follow‐up, and not VHR ASCVD)—referred to as the risk set. From the risk set, a patient with non‐VHR ASCVD was matched to a patient with VHR ASCVD on age (±3 years), sex, and region using a greedy match algorithm.^18^ The matched non‐VHR ASCVD patient was then assigned an index date equivalent to the corresponding case. The process was repeated until all patients with non‐VHR ASCVD were paired with those with VHR ASCVD, resulting in a matched cohort. Patients were excluded from the study if there were missing data (such as age and sex), and if matched non‐VHR ASCVD patients could not be identified using the IDS method.

Patients with VHR ASCVD were further grouped into the following mutually exclusive subgroups in a hierarchical manner based on the major ASCVD event(s) that led to their qualification as VHR ASCVD: (1) patients with ≥2 major ASCVD events; (2) patients with 1 major ASCVD event, which was recent ACS; (3) patients with 1 major ASCVD event, which was a history of MI (non‐recent ACS); (4) patients with 1 major ASCVD event, which was a history of IS; (5) patients with 1 major ASCVD event, which was symptomatic PAD.

In stage 2 of the study, the occurrence of major CV events in the IDS‐matched VHR ASCVD and non‐VHR ASCVD cohorts was estimated (Figure 1). Patients with VHR ASCVD identified in stage 1 of the study were re‐assigned an index date, defined as the first date of their qualifying VHR ASCVD event/risk factor from January 1, 2012, to May 31, 2018. This ensured a ≥1‐year pre‐index period for the assessment of baseline characteristics and a ≥1 month post‐index period for all patients. The matched patients with non‐VHR ASCVD were indexed to the date of the corresponding VHR ASCVD patients' index date. For the assessment of CV events in the 1 and 2 years post‐index, patients were followed‐up for a variable post‐index period from the index date until the end of the study (June 30, 2018), end of the reporting period, or the end of continuous enrollment for pharmacy and medical benefits, whichever came first.

### 2.2 Outcomes

We assessed demographics at index (age, sex, insurance type, and geographical region), current LLT patterns in the 90 days and 1‐year pre‐index period (statin only, statin plus ezetimibe, ezetimibe only), and clinical characteristics in the 1‐year pre‐index period (comorbidities and ASCVD type). Overall cumulative 1‐year and 2‐year rates of the following acute CV events per 100 patient‐years were assessed: MI, IS, unstable angina (UA) hospitalization, coronary revascularization, composite MI/IS, composite MI/IS/UA hospitalization/coronary revascularization. MI, IS, and UA hospitalizations were assessed using IP events (diagnosis at primary position only). Coronary revascularizations were assessed using IP/OP events (diagnosis at any position). The composite MI/IS event rate was assessed using MI/IS events (IP only, diagnosis at primary position). The composite MI/IS/UA hospitalization/coronary revascularization event rate was assessed using MI/IS/UA hospitalization events (IP only, diagnosis at primary position) or revascularization (IP or OP). After observation of the first acute CV event, all subsequent acute CV events of the same type were counted as the same episode if they occurred within 30 days of the discharge date of the previous event. Coronary revascularization occurring within 30 days of discharge from a prior MI/IS/UA hospitalization, or a prior revascularization, was not considered as a distinct event. The CV event rates were reported among all VHR ASCVD patients, all matched non‐VHR ASCVD patients, and VHR ASCVD subgroups.

### 2.3 Ethics

This was a retrospective analysis of de‐identified aggregate claims data; therefore, informed consent, ethics committee approval, or institutional review board approval was not required. The study complied with all applicable laws regarding patient privacy, using Health Insurance Portability and Accountability Act–compliant de‐identified retrospective data sources. No direct patient contact or primary collection of individual human patient data occurred. Study results were in tabular form and aggregate analyses, which omitted patient identification information. All authors had full access to all the data in the study and take responsibility for its integrity and data analysis.

### 2.4 Statistical analysis

Analyses were conducted using SAS version 9.3 (SAS Institute, Cary, NC, USA). The study was descriptive in nature and formal statistical tests for comparison were not conducted. Mean, median, and standard deviation (SD) were generated as a measure of central tendency and variance for continuous variables. For categorical variables, frequencies and percentages were calculated. Overall, major CV event rates were expressed per 100 patient‐years along with 95% confidence intervals (CIs) and calculated using the following formula: number of distinct CV events in all post‐index period × 100/patient‐years from index to the earliest of the following: end of reporting period; end of continuous enrollment; or end of study period (June 30, 2018). Mortality data were unavailable in the linked databases and were not included in the calculation of major CV event rates.

## 3 RESULTS

### 3.1 Baseline demographic and clinical characteristics

Baseline demographic and clinical characteristics are reported in Table 1. The overall study population included 423 632 patients diagnosed with ASCVD, of which 147 679 (34.9%) had ≥1 major ASCVD event. Among patients with ≥1 major ASCVD event, the majority (117 460 [79.5%]) qualified as VHR ASCVD. Similar results were also observed for all major ASCVD patient subgroups: 95% of all patients with ≥1 occurrence of recent ACS, 90% of all those with ≥1 history of MI, 83% of those with ≥1 prior IS, and 95% of those with ≥1 occurrence of symptomatic PAD qualified as VHR ASCVD. Among all patients with ≥1 major ASCVD event, 21.6% qualified as VHR ASCVD through the criteria of ≥2 major ASCVD events, and the remaining 78.4% qualified through other qualifiers of a major ASCVD event and multiple risk factors per 2018 ACC/AHA guideline definition.^7^ A history of IS and hypertension were the most common qualifying major ASCVD event and high‐risk condition, respectively (Table 2). The remaining 306 172 patients from the overall study population were non‐VHR ASCVD patients.

**TABLE 1:** Baseline demographic and clinical characteristics of VHR and non‐VHR ASCVD patients after IDS matching

	IDS‐matched VHR ASCVD (*N* = 115 460)	IDS‐matched non‐VHR ASCVD (*N* = 115 460)
Demographics		
Mean (SD) age, years	60.8 (10.3)	60.9 (10.1)
Men, %	61.1	61.1
Geographic region, %		
Northeast	17.3	17.3
Midwest	11.6	11.6
South	64.3	64.3
West	6.8	6.8
Payer type, %		
Commercial	79.6	80.4
Medicare	16.9	17.4
Other	3.5	2.2
Clinical characteristics		
Mean (SD) baseline LDL‐C, mg/dL	107.0 (39.9)	97.0 (35.0)
Mean (SD) baseline LDL‐C, mmol/L	2.8 (1.0)	2.5 (0.9)
Mean (SD) LDL‐C in patients currently receiving statins and/or ezetimibe, mg/dL	99.8 (39.2)	90.3 (33.5)
Mean (SD) LDL‐C in patients currently receiving statins and/or ezetimibe, mmol/L	2.6 (1.0)	2.3 (0.9)
LDL‐C distribution in patients currently receiving statins and/or ezetimibe		
n	21 153	20 648
LDL‐C <70 mg/dL (<1.8 mmol/L), %	22.5	27.9
LDL‐C 70–99 mg/dL (1.8–2.6 mmol/L), %	32.9	40.2
LDL‐C 100–129 mg/dL (2.6–3.3 mmol/L), %	25.1	19.9
LDL‐C 130–159 mg/dL (3.4–4.1 mmol/L), %	11.7	8.0
LDL‐C 160–189 mg/dL (4.1–4.9 mmol/L), %	4.9	2.8
LDL‐C >189 mg/dL (>4.9 mmol/L), %	2.9	1.2
ASCVD type, %		
MI	56.3	8.4
UA hospitalization	3.4	0.3
Stable angina hospitalization	7.5	5.1
IS	37.4	11.4
TIA	13.9	13.2
PCI	26.7	14.2
CABG	11.1	8.7
PAD	20.4	28.3
Other ASCVD	53.2	64.2
LLT use (1‐year pre‐index), %		
Any statin and/or ezetimibe	59.0	60.5
Statin only	55.0	55.8
High‐intensity statin	17.8	17.8
Medium‐intensity statin	31.8	32.9
Low‐intensity statin	5.4	5.2
Statin + ezetimibe	3.3	4.0
High‐intensity statin	1.4	1.8
Medium‐intensity statin	1.6	2.0
Low‐intensity statin	0.2	0.2
Ezetimibe only	0.6	0.7
Current LLT use (90 days pre‐index), %		
Any statin and/or ezetimibe	51.7	52.7
Statin only	48.7	49.1
High‐intensity statin	16.2	16.0
Medium‐intensity statin	27.9	28.7
Low‐intensity statin	4.6	4.4
Statin + ezetimibe	2.4	2.9
High‐intensity statin	1.1	1.3
Medium‐intensity statin	1.2	1.5
Low‐intensity statin	0.1	0.1
Ezetimibe only	0.6	0.7

Abbreviations: ASCVD, atherosclerotic cardiovascular disease; CABG, coronary artery bypass graft; IDS, incidence density sampling; IS, ischemic stroke; LDL‐C, low‐density lipoprotein cholesterol; LLT, lipid‐lowering therapy; MI, myocardial infarction; PAD, peripheral arterial disease; PCI, percutaneous coronary intervention; SD, standard deviation; TIA, transient ischemic attack; UA, unstable angina; VHR, very high‐risk.

**TABLE 2:** Proportions of qualifying major ASCVD events and high‐risk conditions from the 2018 ACC/AHA blood cholesterol guideline

	IDS‐matched VHR ASCVD (*N* = 115 460)	IDS‐matched non‐VHR ASCVD (*N* = 115 460)
Major ASCVD event(s), %		
≥2 major ASCVD events	18.6	0.0
Recent ACS	28.8	1.4
History of MI (other than recent ACS)	30.4	7.7
History of IS	35.7	6.9
Symptomatic PAD	8.5	1.5
High‐risk conditions, %		
Hypertension	84.3	69.5
Diabetes mellitus	43.0	32.6
Age ≥65 years	34.5	32.9
History of prior CABG/PCI	27.6	17.7
Current smoking	25.7	14.8
Persistently elevated LDL‐C (≥100 mg/dL [≥2.6 mmol/L]) despite statin and/or ezetimibe	19.5	15.7
History of CHF	14.6	8.9
CKD	5.6	4.3
HeFH	2.2	1.3

Abbreviations: ACC, American College of Cardiology; ACS, acute coronary syndrome; AHA, American Heart Association; ASCVD, atherosclerotic cardiovascular disease; CABG, coronary artery bypass graft; CHF, congestive heart failure; CKD, chronic kidney disease; HeFH, heterozygous familial hypercholesterolemia; IDS, incidence density sampling; IS, ischemic stroke; LDL‐C, low‐density lipoprotein cholesterol; MI, myocardial infarction; PAD, peripheral arterial disease; PCI, percutaneous coronary intervention; VHR, very high‐risk.

After IDS matching, there were 115 460 patients each in IDS‐matched VHR and non‐VHR ASCVD cohorts. The mean age of the patients was 61 years, 61% were men, and 80% had commercial insurance (Table 1). The differences in the baseline demographic and clinical characteristics of the VHR and non‐VHR ASCVD cohorts before IDS matching are listed in Supplementary Table 3.

For current LLT (90‐days pre‐index), 51.7% of the VHR ASCVD cohort was treated with statins and/or ezetimibe; 16.2% and 27.9% of patients were prescribed high‐intensity and medium‐intensity statin monotherapy, respectively. In the non‐VHR ASCVD cohort, 52.7% of patients were treated with statins and/or ezetimibe; 16.0% and 28.7% of patients were prescribed high‐intensity and medium‐intensity statin monotherapy, respectively. Low numbers of patients in both cohorts were treated with ezetimibe only (Table 1). In the expanded look‐back period of 1‐year pre‐index, current LLT use increased to only 59.0% of patients in the VHR ASCVD cohort (Table 1).

The mean (SD) baseline LDL‐C was 107.0 (39.9) mg/dL (2.8 [1.0] mmol/L) and 97.0 (35.0) mg/dL (2.5 [0.9] mmol/L) in the VHR and non‐VHR ASCVD cohorts, respectively. In patients treated with statins and/or ezetimibe in the VHR (*n* = 21 153) and non‐VHR (*n* = 20 648) ASCVD cohorts, the mean (SD) LDL‐C was 99.8 (39.2) mg/dL (2.6 [1.0] mmol/L) and 90.3 (33.5) mg/dL (2.3 [0.9] mmol/L), respectively (Table 1). Despite current statin and/or ezetimibe use, 77.5% and 72.1% of patients in the VHR and non‐VHR ASCVD cohorts, respectively, had LDL‐C ≥70 mg/dL (≥1.8 mmol/L), with a similar LDL‐C distribution between cohorts (Table 1).

### 3.2 Overall CV event rates post‐index

**FIGURE 2:**
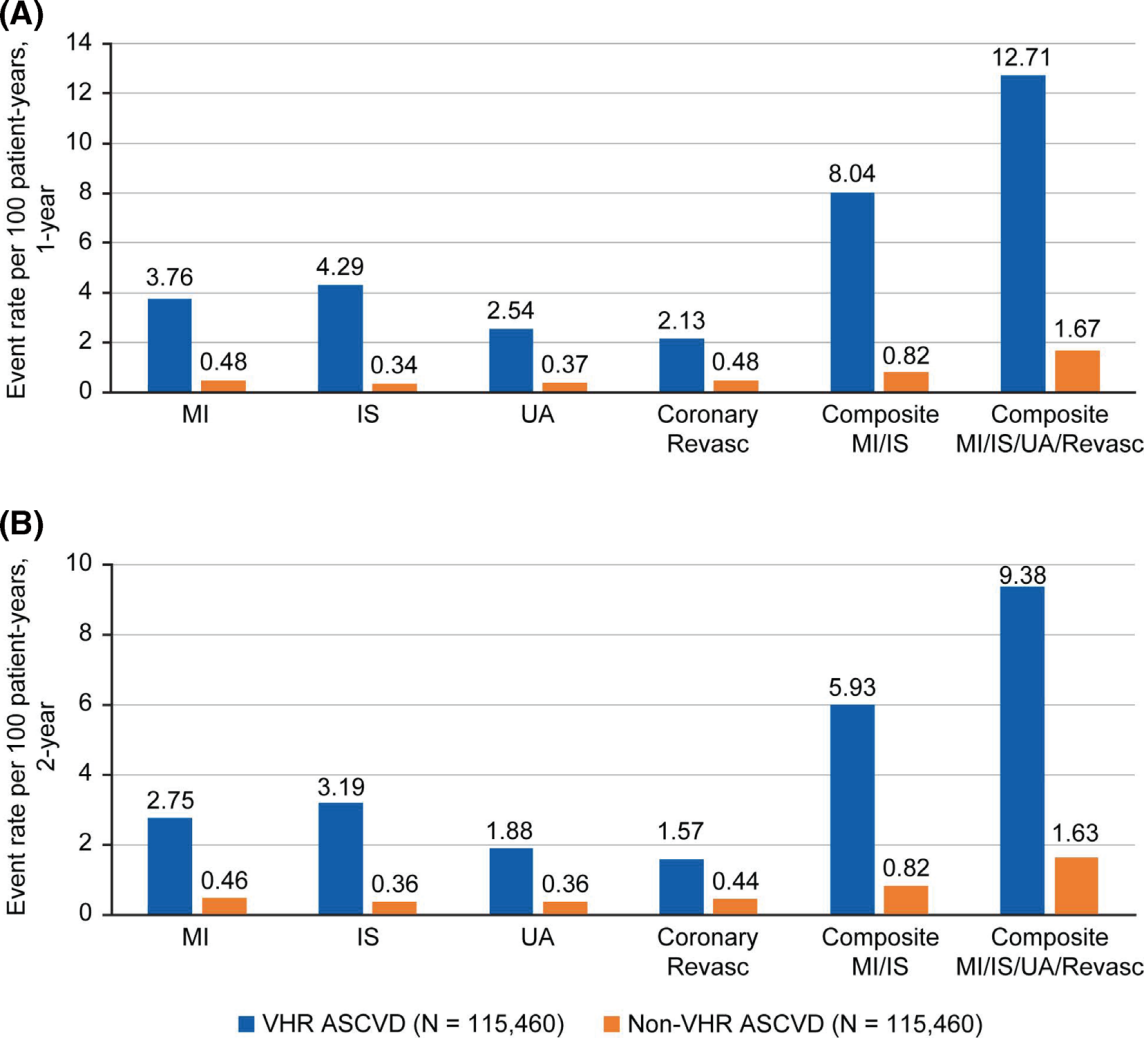
Overall rates of major CV events per 100 patient‐years in IDS‐matched VHR and non‐VHR ASCVD cohorts for (A) 1‐year post‐index and (B) 2‐years post‐index. ASCVD, atherosclerotic cardiovascular disease; CV, cardiovascular; IDS, incidence density sampling; IS, ischemic stroke; MI, myocardial infarction; Revasc, revascularization; UA, unstable angina hospitalization; VHR, very high‐risk

In the VHR ASCVD cohort, the median (interquartile range [IQR]) follow‐up times were 365 (0) days and 730 (314) days for the assessment of 1‐year and 2‐year CV event rates, respectively. In the non‐VHR ASCVD cohort, the median (IQR) follow‐up times were 365 (0) days and 730 (121) days, respectively. The overall composite MI/IS event rates for patients with VHR ASCVD during the 1‐year and 2‐year post‐index period were 8.04 (95% CI: 7.87–8.22) and 5.93 (95% CI: 5.82–6.05) events per 100 patient‐years, respectively (Figure 2; Table 3). The overall composite MI/IS event rates for IDS‐matched patients with non‐VHR ASCVD during the 1‐year and 2‐year post‐index period were 0.82 (95% CI: 0.77–0.88) and 0.82 (95% CI: 0.78–0.86) events per 100 patient‐years, respectively (Figure 2; Table 3). When UA hospitalization and coronary revascularization were also included, the composite CV event rates during the 1‐year and 2‐year post‐index period were 12.71 (95% CI: 12.50–12.93) and 9.38 (95% CI: 9.24–9.53) events per 100 patient‐years, respectively, for patients with VHR ASCVD; and 1.67 (95% CI: 1.60–1.75) and 1.63 (95% CI: 1.57–1.68) events per 100 patient‐years, respectively, for patients with non‐VHR ASCVD (Figure 2; Table 3). Major CV event rates defined by the VHR ASCVD subgroups in the 1‐year post‐index period are summarized in Table 3, with the highest composite CV event rate observed in patients with recent ACS: 19.20 (95% CI: 18.64–19.78) events per 100 patient‐years.

**TABLE 3:** Overall rates of major CV events per 100 patient‐years by VHR ASCVD subgroups (1‐year post‐index)

Event rate per 100 patient‐years, (95% CI)	IDS‐matched VHR ASCVD (*N* = 115 460)	IDS‐matched non‐VHR ASCVD (*N* = 115 460)	Multiple events (*N* = 21 450)	Recent ACS (*N* = 25 218)	History of MI (*N* = 29 054)	History of IS (*N* = 31 816)	Symptomatic PAD (*N* = 7922)
MI	3.76 (3.64–3.88)	0.48 (0.44–0.52)	3.50 (3.24–3.78)	8.09 (7.72–8.47)	3.89 (3.65–4.13)	0.93 (0.82–1.05)	1.32 (1.08–1.61)
IS	4.29 (4.16–4.41)	0.34 (0.31–0.38)	5.68 (5.35–6.03)	1.52 (1.37–1.69)	0.62 (0.53–0.73)	9.81 (9.45–10.18)	1.04 (0.83–1.31)
UA hospitalization	2.54 (2.44–2.64)	0.37 (0.33–0.41)	3.32 (3.07–3.59)	6.24 (5.93–6.58)	1.45 (1.31–1.61)	0.47 (0.40–0.56)	0.72 (0.55–0.95)
Coronary revascularization	2.13 (2.04–2.22)	0.48 (0.44–0.53)	1.71 (1.53–1.90)	3.34 (3.11–3.59)	3.14 (2.93–3.36)	0.66 (0.57–0.76)	1.50 (1.24–1.81)
Composite MI/IS	8.04 (7.87–8.22)	0.82 (0.77–0.88)	9.18 (8.75–9.62)	9.61 (9.21–10.02)	4.51 (4.26–4.78)	10.74 (10.36–11.13)	2.36 (2.03–2.74)
Composite MI/IS/UA hospitalization/coronary revascularization	12.71 (12.50–12.93)	1.67 (1.60–1.75)	14.20 (13.68–14.75)	19.20 (18.64–19.78)	9.10 (8.73–9.47)	11.87 (11.47–12.28)	4.58 (4.11–5.10)

Abbreviations: ACS, acute coronary syndrome; ASCVD, atherosclerotic cardiovascular disease; CI, confidence interval; CV, cardiovascular; IDS, incidence density sampling; IS, ischemic stroke; MI, myocardial infarction; PAD, peripheral arterial disease; UA, unstable angina; VHR, very high‐risk.

## 4 DISCUSSION

Informed by the introduction of the VHR ASCVD criteria in the ACC/AHA 2018 guideline for the management of blood cholesterol,^7^ this observational retrospective cohort study from routine clinical practice provides new and important data on the clinical characteristics, treatment patterns, and risk of major CV events in patients with VHR versus non‐VHR ASCVD treated in US healthcare settings. At the time of writing, this study is the second to operationalize the 2018 ACC/AHA VHR ASCVD criteria in real‐world clinical practice and assess major CV event rates in this patient population. The incidence of VHR ASCVD was relatively common, with the majority of patients (80%) with ≥1 major ASCVD event meeting the ACC/AHA VHR ASCVD criteria,^7^ suggesting that most patients with ≥1 major ASCVD event will require intensive LDL‐C lowering. Despite clinical guidelines recommending that all patients with ASCVD take a high‐intensity statin or the maximally tolerated statin dosage,^7^ only 51.7% of patients with VHR ASCVD received LLT with a statin and/or ezetimibe, and most of these patients still had suboptimally controlled LDL‐C ≥70 mg/dL (≥1.8 mmol/L). Moreover, despite the same proportion of statin and/or ezetimibe utilization, mean LDL‐C was higher in patients with VHR ASCVD versus patients with non‐VHR ASCVD, reinforcing the unmet need for improved LDL‐C control in patients at the highest level of CV risk. Previous studies have reported low utilization and adherence to LLT in patients with ASCVD treated in the US.^19^, ^20^, ^21^, ^22^, ^23^ For example, an analysis from the GOULD (Getting to an Improved Understanding of Low‐Density Lipoprotein Cholesterol and Dyslipidemia Management) registry of patients with ASCVD reports that high‐intensity statins and ezetimibe were utilized in only 44% and 9% of patients, respectively.^23^ Notably, the current study represents a new addition to the existing literature, by describing real‐world treatment patterns specifically in patients meeting the ACC/AHA 2018 guideline criteria for VHR ASCVD.^7^

Overall, major CV event rates were high in patients with VHR ASCVD, with a rate of composite MI/IS events approximately 10 times greater than in patients with non‐VHR ASCVD in the 1‐year post‐index period. However, it is important to note that patients with non‐VHR ASCVD were still at considerable risk of major CV events. The results provide support for the validity of the VHR ASCVD category as a means of identifying patients who may receive substantial benefit—in terms of absolute risk reduction—through intensive LLT, including with non‐statin therapies such as PCSK9 inhibitors.^6^, ^8^, ^9^ With regard to contextualizing the current results within the literature, a recent study estimated ASCVD event rates among 27 778 adults in the MarketScan health insurance database with a history of ASCVD who met and did not meet the definition of VHR ASCVD per the 2018 ACC/AHA cholesterol guideline.^7^, ^24^ The rate of major CV events was higher in patients with VHR versus non‐VHR ASCVD (53.1 vs. 17 per 1000 patient‐years, respectively).^24^ Of note, the major CV event rates observed in the current study were higher than those in the analysis of the MarketScan health insurance database.^24^ This may be accounted for by differences in methodology as in the current study, patients were followed‐up immediately after they qualified as VHR ASCVD; thus, there was higher CV risk captured in the current analysis than in the previous analysis of the MarketScan health insurance database.^24^

Broadly, this study demonstrates that patients with ASCVD were exposed to a high residual CV risk due to suboptimally controlled LDL‐C above ACC/AHA 2018 guideline recommendations,^7^ indicating an unmet treatment need. Increasing the rate and intensity of LLT in this population via optimization of statin therapy, as well as greater use of non‐statin therapy when needed, in accordance with ACC/AHA 2018 guidelines,^7^ has the potential to reduce major CV event rates and improve patient outcomes.^4^, ^5^, ^6^, ^8^ Indeed, it is now recognized that patients with the highest levels of CV risk derive greater absolute and relative risk reductions with the addition of non‐statin therapies (e.g., ezetimibe and PCSK9 inhibitors) than those with lower CV risk.^6^, ^8^, ^9^, ^25^ Moreover, the value of PCSK9 inhibitors is improved by selecting patients at higher risk for the occurrence of CV events,^26^ and the major CV event rates observed in the current study were within the range where the addition of PCSK9 inhibitors to background LLT would meet cost‐effectiveness thresholds from models based on ACC/AHA guidelines.^7^, ^26^

The results of this study should be considered within the context of several limitations. First, it was a retrospective analysis using linked commercial claims databases and was therefore subject to the inherent limitations of this methodology, including limited generalizability to patients without commercial insurance and to those aged ≥65 years. Second, there was a lack of CV mortality data, a limitation well‐recognized for US‐based claims datasets, and this information was therefore not included in the assessment of major CV events. However, this limitation would be expected to result in an underestimation, rather than an overestimation, of CV events during follow‐up. Third, as we translated the ACC/AHA 2018 VHR ASCVD criteria into claims data variables, coding errors could have led to a misclassification of patients' disease state; however, the validity of using billing codes to identify various CV events has been previously established.^27^, ^28^ Fourth, the IDS methodology may still have resulted in meaningful differences between matched patients with ASCVD being compared, beyond the VHR categorization.

In conclusion, the majority of patients with ≥1 previous major ASCVD event qualified as VHR ASCVD in real‐world US clinical practice. In patients with VHR ASCVD, LLT utilization rates were relatively low and LDL‐C was suboptimally controlled, even in patients receiving high‐intensity statins and/or ezetimibe treatment. Application of the ACC/AHA 2018 guideline VHR ASCVD criteria was able to identify patients with higher rates of overall major CV events (compared with those not meeting the VHR ASCVD criteria), who would therefore derive the greatest absolute benefit from more intensive LDL‐C lowering.

## CONFLICT OF INTEREST

GCF reports consulting for Abbott, Amgen, AstraZeneca, Bayer, Janssen, Merck, and Novartis. MNK is a consultant for Vifor Pharma and reports personal fees from AstraZeneca; grants, personal fees, and other from AstraZeneca; grants and personal fees from Boehringer Ingelheim; and personal fees from Sanofi, Amgen, Novo Nordisk, Merck (Diabetes), Janssen, Bayer, GlaxoSmithKline, Glytec, Novartis, Applied Therapeutics, Amarin, and Eli Lilly. PBR, GV, MH, JA, and KEM were employees and stockholders of Amgen at the time of the study. SN, KS, and RLW were employees of IQVIA at the time of the study, which received consulting fees from Amgen to conduct the study.

## Supporting information


**Supplementary Table 1** ASCVD diagnosis codes
Supplementary Table 2. Definition of major ASCVD events and high‐risk conditions in the 2018 ACC/AHA blood cholesterol guideline
Supplementary Table 3. Baseline demographics and clinical characteristics in VHR and non‐VHR ASCVD cohorts before IDS matching

## Data Availability

Qualified researchers may request data from Amgen clinical studies. Complete details are available at the following: http://www.amgen.com/datasharing.
