# Supplementary material for: Patient characteristics and acute cardiovascular event rates among patients with very high‐risk and non‐very high‐risk atherosclerotic cardiovascular disease
Source: Clin Cardiol. 2021 Aug 5;44(10):1457–66. doi: 10.1002/clc.23706 (PMC8495090; doi:10.1002/clc.23706)
Supplement: Supplementary file 1 — Supplementary Table 1 ASCVD diagnosis codes Supplementary Table 2. Definition of major ASCVD events and high‐risk conditions in the 2018 ACC/AHA blood cholesterol guideline Supplementary Table 3. Baseline demographics and clinical characteristics in VHR and non‐VHR ASCVD cohorts before IDS matching [file CLC-44-1457-s001.docx]

Supplementary material

Supplementary Table 1. ASCVD diagnosis codes

| ASCVD diagnosis | Codes |
| --- | --- |
| Myocardial infarction | **ICD-10-CM**: I21.01, I21.02, I21.09, I21.11, I21.21, I21.29, I21.3, I21.4, I22.0, I22.1, I22.2, I22.8, I22.9, I25.2, I23.0, I23.1, I23.2, I23.3, I23.4, I23.5, I23.6, I23.7, I23.8  **ICD-9**: 410.00, 410.01, 410.02, 410.10, 410.11, 410.12, 410.20, 410.21, 410.22, 410.30, 410.31, 410.32, 410.40, 410.41, 410.42, 410.50, 410.51, 410.52, 410.60, 410.61, 410.62, 410.70, 410.71, 410.72, 410.80, 410.81, 410.82, 410.90, 410.91, 410.92, 412, 429.79, 411.0 |
| Unstable angina | **ICD-10-CM**: I20.0, I20.1, I20.8, I20.9, I23.7, I24.0, I24.8, I25.110, I25.111, I25.118, I25.119, I25.700, I25.701, I25.708, I25.709, I25.710, I25.711, I25.718, I25.719, I25.720, I25.721, I25.728, I25.729, I25.730, I25.731, I25.738, I25.739, I25.750, I25.751, I25.758, I25.759, I25.760, I25.761, I25.768, I25.769, I25.790, I25.791, I25.798, I25.799  **ICD-9**: 411.1, 411.81, 411.89, 413.9, 413.0 |
| Ischemic stroke | **ICD-10-CM:** Z86.73, I63.00, I63.011, I63.012, I63.013, I63.019, I63.02, I63.031, I63.032, I63.033, I63.039, I63.09, I63.20, I63.211, I63.212, I63.213, I63.219, I63.22, I63.231, I63.232, I63.233, I63.239, I63.29, I63.30, I63.311, I63.312, I63.313, I63.319, I63.321, I63.322, I63.323, I63.329, I63.331, I63.332, I63.333, I63.339, I63.341, I63.342, I63.343, I63.349, I63.39, I63.50, I63.511, I63.512, I63.513, I63.519, I63.521, I63.522, I63.523, I63.529, I62.531, I63.532, I63.533, I63.539, I63.541, I63.542, I63.543, I63.549, I63.59, I63.6, I63.8, I63.9  **ICD-9-CM**: 434.01, 434.91, 433.01, 433.11, 433.21, 433.31, 433.81, 433.91, V12.54, 436 |
| Transient ischemic attack | **ICD-10-CM**: G45.0, G45.1, G45.2, G45.3, G45.8, G45.9, G46.0, G46.1, H34.00, H34.01, H34.02, H34.03, I67.841, I67.848  **ICD-9-CM**: 362.34, 435.0, 435.1, 435.2, 435.3, 435.8, 435.9 |
| Coronary revascularization (percutaneous coronary intervention) | **ICD-10-CM**: Z98.61, Z95.5, T82.855A, T82.855D, T82.855S  **ICD-10-PCS**: 0270346, 027034Z, 0270356, 027035Z, 0270366, 027036Z, 0270376, 027037Z, 02703D6, 02703DZ, 02703E6, 02703EZ, 02703F6, 02703FZ, 02703G6, 02703GZ, 02703T6, 02703TZ, 02703Z6, 02703ZZ, 0270446, 027044Z, 0270456, 027045Z, 0270466, 027046Z, 0270476, 027047Z, 02704D6, 02704DZ, 02704E6, 02704EZ, 2704F6, 02704FZ, 02704G6, 02704GZ, 02704T6, 02704TZ, 02704Z6, 02704ZZ, 0271346, 027134Z, 0271356, 027135Z, 0271366, 027136Z, 0271376, 027137Z, 02713D6, 02713DZ, 02713E6, 02713EZ, 02713F6, 02713FZ, 02713G6, 02713GZ, 02713T6, 02713TZ, 02713Z6, 02713ZZ, 0271446, 027144Z, 0271456, 027145Z, 0271466, 027146Z, 0271476, 027147Z, 02714D6, 02714DZ, 02714E6, 02714EZ, 02714F6, 02714FZ, 02714G6, 02714GZ, 02714T6, 02714TZ, 02714Z6, 02714ZZ, 0272346, 027234Z, 0272356, 027235Z, 0272366, 027236Z, 0272376, 027237Z, 02723D6, 02723DZ, 02723E6, 02723EZ, 02723F6, 02723FZ, 02723G6, 02723GZ, 02723T6, 02723TZ, 02723Z6, 02723ZZ, 0272446, 027244Z, 0272456, 027245Z, 0272466, 027246Z, 0272476, 027247Z, 02724D6, 02724DZ, 02724E6, 02724EZ, 02724F6, 02724FZ, 02724G6,02724GZ, 02724T6, 02724TZ, 02724Z6, 02724ZZ, 0273346, 027334Z, 0273356, 027335Z, 0273366, 027336Z, 0273376, 027337Z, 02733D6, 02733DZ, 02733E6, 02733EZ, 02733F6, 02733FZ, 02733G6, 02733GZ, 02733T6, 02733TZ, 02733Z6, 02733ZZ, 0273446, 027344Z, 0273456, 027345Z, 0273466, 027346Z, 0273476, 027347Z, 02734D6, 02734DZ, 02734E6, 02734EZ, 02734F6, 02734FZ, 02734G6, 02734GZ, 02734T6, 02734TZ, 02734Z6, 02734ZZ, 02C03ZZ, 02C04ZZ, 02C13ZZ, 02C14ZZ, 02C23ZZ, 02C24ZZ, 02C33ZZ, 02C34ZZ, X2C0361, X2C1361, X2C2361, X2C3361  **CPT/****HCPCS:** 92973, C9600, C9601, 92920, 92921, 92928, 92929, 92924, 92925, 92933, 92934, 92937, 92938, 92941, 92943, 92944, C9602, C9603, C9604, C9605, C9606, C9607, C9608, 92975, 92977  **ICD-9:** V45.82, 00.66, 1755, 36.06, 36.07, 00.40, 00.41, 00.42, 00.43, 00.44, 00.45, 00.46, 00.47, 00.48 |
| Coronary revascularization (coronary artery bypass grafting) | **ICD-10-CM**: Z95.1  **ICD-10-PCS**: 210083, 0210088, 0210089, 021008C, 021008F, 021008W, 0210093, 0210098, 0210099, 021009C, 021009F, 021009W, 02100A3, 02100A8, 02100A9, 02100AC, 02100AF, 02100AW, 02100J3,02100J8, 02100J9, 02100JC, 02100JF, 02100JW, 02100K3, 02100K8, 02100K9, 02100KC, 02100KF, 02100KW, 02100Z3, 02100Z8, 02100Z9, 02100ZC, 02100ZF, 0210344, 02103D4, 0210444, 0210483, 0210488, 0210489, 021048C, 021048F, 021048W, 0210493, 0210498, 0210499, 021049C, 021049F, 021049W, 02104A3, 02104A8, 02104A9, 02104AC, 02104AF, 02104AW, 02104D4, 02104J3,02104J8, 02104J9, 02104JC, 02104JF, 02104JW, 02104K3, 02104K8, 02104K9, 02104KC, 02104KF, 02104KW, 02104Z3, 02104Z8, 02104Z9, 02104ZC, 02104ZF, 0211083, 0211088, 0211089, 021108C, 021108F, 021108W, 0211093, 0211098, 0211099, 021109C, 021109F, 021109W, 02110A3, 02110A8, 02110A9, 02110AC, 02110AF, 02110AW, 02110J3, 02110J8, 02110J9, 02110JC, 02110JF, 02110JW, 02110K3, 02110K8, 02110K9, 02110KC, 02110KF, 02110KW, 02110Z3, 02110Z8, 02110Z9, 02110ZC, 02110ZF, 0211344, 02113D4, 0211444, 0211483, 0211488, 0211489, 021148C, 021148F, 021148W, 0211493, 0211498, 0211499, 021149C, 021149F, 021149W, 02114A3, 02114A8, 02114A9, 02114AC, 02114AF, 02114AW, 02114D4, 02114J3, 02114J8, 02114J9, 02114JC, 02114JF, 02114JW, 02114K3, 02114K8, 02114K9, 02114KC, 02114KF, 02114KW, 02114Z3, 02114Z8, 02114Z9, 02114ZC, 02114ZF, 0212083, 0212088, 0212089, 021208C, 021208F, 021208W, 0212093, 0212098, 0212099, 021209C, 021209F, 021209W, 02120A3, 02120A8, 02120A9, 02120AC, 02120AF, 02120AW, 02120J3, 02120J8, 02120J9, 02120JC, 02120JF, 02120JW, 02120K3, 02120K8, 02120K9, 02120KC, 02120KF, 02120KW, 02120Z3, 02120Z8, 02120Z9, 02120ZC, 02120ZF, 0212344, 02123D4, 0212444, 0212483, 0212488, 0212489, 021248C, 021248F, 021248W, 0212493, 0212498, 0212499, 021249C, 021249F, 021249W, 02124A3, 02124A8, 02124A9, 02124AC, 02124AF, 02124AW, 02124D4, 02124J3, 02124J8, 02124J9, 02124JC, 02124JF, 02124JW, 02124K3, 02124K8, 02124K9, 02124KC, 02124KF, 02124KW, 02124Z3, 02124Z8, 02124Z9, 02124ZC, 02124ZF, 0213083, 0213088, 0213089, 021308C, 021308F, 021308W, 0213093, 0213098, 0213099, 021309C, 021309F, 021309W, 02130A3, 02130A8, 02130A9, 02130AC, 02130AF, 02130AW, 02130J3, 02130J8, 02130J9, 02130JC, 02130JF, 02130JW, 02130K3, 02130K8, 02130K9, 02130KC, 02130KF, 02130KW, 02130Z3, 02130Z8, 02130Z9, 02130ZC, 02130ZF, 0213344, 02133D4, 0213444, 0213483, 0213488, 0213489, 021348C, 021348F, 021348W, 0213493, 0213498, 0213499, 021349C, 021349F, 021349W, 02134A3, 02134A8, 02134A9, 02134AC, 02134AF, 02134AW, 02134D4, 02134J3, 02134J8, 02134J9, 02134JC, 02134JF, 02134JW, 02134K3, 02134K8, 02134K9, 02134KC, 02134KF, 02134KW, 02134Z3, 02134Z8, 02134Z9, 02134ZC, 02134ZF  **CPT/HCPCS:** 33510, 33511, 33512, 33513, 33514, 33516, 33517, 33518, 33519, 33521, 33522, 33523, 33533, 33534, 33535, 33536, 33508, S2205, S2206, S2207, S2208, S2209  **ICD-9-CM:** V45.81, 361, 36.10, 36.11, 36.12, 36.13, 36.14, 36.15, 36.16, 36.17, 36.19, 362, 363, 36.31, 36.32, 36.33, 36.34, 36.39 |
| Coronary revascularization (other) | **ICD-10-PCS**: 0270046, 027004Z, 02700D6, 02700DZ, 02700T6, 02700TZ, 02700Z6, 02700ZZ, 0271046, 027104Z, 02710D6, 02710DZ, 02710T6, 02710TZ, 02710Z6, 02710ZZ, 0272046, 027204Z, 02720D6, 02720DZ, 02720T6, 02720TZ, 02720Z6, 02720ZZ, 0273046, 027304Z, 02730D6, 02730DZ, 02730T6, 02730TZ, 02730Z6, 02730ZZ, 02C00ZZ, 02C10ZZ, 02C20ZZ, 02C30ZZ  **ICD-9-CM**: 36.03, 36.04, 36.09  **CPT**: 33140, 33141, 33572 |
| Peripheral arterial disease | **ICD-10-CM:** I74.01, I74.09, I74.3, I74.5, Z95.820, Z98.62 Z95.828, I70.1, I70.291, I70.292, I70.293, I70.298, I70.299, I70.401, I70.402, I70.403, I70.408, I70.409, I70.491, I70.492, I70.493, I70.498, I70.499, I70.501, I70.502, I70.503, I70.508, I70.509, I70.591, I70.592, I70.593, I70.598, I70.599, I70.92, I70.8, I70.90, I70.91, I75.011, I75.012, I75.013, I75.019, I75.021, I75.022, I75.023, I75.029, I75.81, I75.89, I70.0, I70.201, I70.202, I70.203, I70.208, I70.209, I70.301, I70.302, I70.303, I70.308, I70.309, I70.391, I70.392, I70.393, I70.398, I70.399, I70.601, I70.602, I70.603, I70.608, I70.609, I70.691, I70.692, I70.693, I70.698, I70.699, I70.701, I70.702, I70.703, I70.708, I70.709, I70.791, I70.792, I70.793, I70.798, I70.799, I73.9, I73.1, I73.89  **ICD-10-PCS:** 3E03317, 3E04317, 3E05317, 3E06317, 3E08317, 0Y6M0Z0, 0Y6N0Z0, 0Y6H0Z3, 0Y6J0Z3, 0Y670ZZ, 0Y680ZZ, 0Y6C0Z1, 0Y6C0Z3, 0Y6D0Z1, 0Y6D0Z2, 0Y6D0Z3, 0Y6F0ZZ, 0Y6G0ZZ, 0Y6H0Z1, 0Y6H0Z2, 0Y6H0Z3, 0Y6J0Z1, 0Y6J0Z2, 0Y6J0Z3, 0Y620ZZ, 0Y630ZZ, 0Y640ZZ, 0Y670ZZ, 0Y680ZZ, 04CC0ZZ, 04CC3ZZ, 04CC4ZZ, 04CD0ZZ, 04CD3ZZ, 04CD4ZZ, 04CE0ZZ, 04CE3ZZ, 04CE4ZZ, 04CF0ZZ, 04CF3ZZ, 04CF4ZZ, 04CH0ZZ, 04CH3ZZ, 04CH4ZZ, 04CJ0ZZ, 04CJ3ZZ, 04CJ4ZZ, 04CK0ZZ, 04CK0Z6, 04CK0ZZ, 04CK4Z6, 04CK4ZZ, 04CL0Z6, 04CL0ZZ, 04CL4Z6, 04CL4ZZ, 04CM0Z6, 04CM0ZZ, 04CM4Z6, 04CM4ZZ, 04CN0Z6, 04CN0ZZ, 04CN4Z6, 04CN4ZZ, 04CP0Z6, 04CP0ZZ, 04CP4Z6, 04CP4ZZ, 04CQ0Z6, 04CQ0ZZ, 04CQ4Z6, 04CQ4ZZ, 04CR0Z6, 04CR0ZZ, 04CR4Z6, 04CR4ZZ, 04CS0Z6, 04CS0ZZ, 04CS4Z6, 04CS4ZZ, 04CT0Z6, 04CT0ZZ, 04CT4Z6, 04CT4ZZ, 04CU0Z6, 04CU0ZZ, 04CU4Z6, 04CU4ZZ, 04CV0Z6, 04CV0ZZ, 04CV4Z6, 04CV4ZZ, 04CW0Z6, 04CW0ZZ, 04CW4Z6, 04CW4ZZ, 04CY0Z6, 04CY0ZZ, 04CY4Z6, 04CY4ZZ, 04HY02Z, 04HY42Z, 04PY0YZ, 04PY3YZ, 04PY4YZ, 04WY0YZ, 04WY3YZ, 04WY4YZ, 04CK3ZZ, 04CK4ZZ, 04CL0ZZ, 04CL3ZZ, 04CL4ZZ, 04CM0ZZ, 04CM3ZZ, 04CM4ZZ, 04CN0ZZ, 04CN3ZZ, 04CN4ZZ, 04CP0ZZ, 04CP3ZZ, 04CP4ZZ, 04CQ0ZZ, 04CQ3ZZ, 04CQ4ZZ, 04CR0ZZ, 04CR3ZZ, 04CR4ZZ, 04CS0ZZ, 04CS3ZZ, 04CS4ZZ, 04CT0ZZ, 04CT3ZZ, 04CT4ZZ, 04CU0ZZ, 04CU3ZZ, 04CU4ZZ, 04CV0ZZ, 04CV3ZZ, 04CV4ZZ, 04CW0ZZ, 04CW3ZZ, 04CW4ZZ, 04CY0ZZ, 04CY3ZZ, 04CY4ZZ, 04RK07Z, 04RK0JZ 04RK0KZ, 04RK47Z, 04RK4JZ, 04RK4KZ, 04RL07Z, 04RL0JZ, 04RL0KZ, 04RL47Z, 04RL4JZ, 04RL4KZ, 04RM07Z, 04RM0JZ, 04RM0KZ, 04RM47Z, 04RM4JZ, 04RM4KZ, 04RN07Z, 04RN0JZ, 04RN0KZ, 04RN47Z, 04RN4JZ, 04RN4KZ, 04RP07Z, 04RP0JZ, 04RP0KZ, 04RP47Z, 04RP4JZ, 04RP4KZ, 04RQ07Z, 04RQ0JZ, 04RQ0KZ, 04RQ47Z, 04RQ4JZ, 04RQ4KZ, 04RR07Z, 04RR0JZ, 04RR0KZ, 04RR47Z, 04RR4JZ, 04RR4KZ, 04RS07Z, 04RS0JZ, 04RS0KZ, 04RS47Z, 04RS4JZ, 04RS4KZ, 04RT07Z, 04RT0JZ, 04RT0KZ, 04RT47Z, 04RT4JZ, 04RT4KZ, 04RU07Z, 04RU0JZ, 04RU0KZ, 04RU47Z, 04RU4JZ, 04RU4KZ, 04RV07Z, 04RV0JZ, 04RV0KZ, 04RV47Z, 04RV4JZ, 04RV4KZ, 04RW07Z, 04RW0JZ, 04RW0KZ, 04RW47Z, 04RW4JZ, 04RW4KZ, 04RY07Z, 04RY0JZ, 04RY0KZ, 04RY47Z, 04RY4JZ, 04RY4KZ, 04BK0ZZ, 04BK3ZZ, 04BK4ZZ, 04BL0ZZ, 04BL3ZZ, 04BL4ZZ, 04BM0ZZ, 04BM3ZZ, 04BM4ZZ, 04BN0ZZ, 04BN3ZZ, 04BN4ZZ, 04BP0ZZ, 04BP3ZZ, 04BP4ZZ, 04BQ0ZZ, 04BQ3ZZ, 04BQ4ZZ, 04BR0ZZ, 04BR3ZZ, 04BR4ZZ, 04BS0ZZ, 04BS3ZZ, 04BS4ZZ, 04BT0ZZ, 04BT3ZZ, 04BT4ZZ, 04BU0ZZ, 04BU3ZZ, 04BU4ZZ, 04BV0ZZ, 04BV3ZZ, 04BV4ZZ, 04BW0ZZ, 04BW3ZZ, 04BW4ZZ, 04BY0ZZ, 04BY3ZZ, 04BY4ZZ, 0410096, 0410097, 0410098, 0410099, 041009B, 041009C, 041009D, 041009F, 041009G, 041009H, 041009J, 041009K, 041009Q, 041009R, 04100A6, 04100A7, 04100A8, 04100A9, 04100AB, 04100AC, 04100AD, 04100AF, 04100AG, 04100AH, 04100AJ, 04100AK, 04100AQ, 04100AR, 04100J6, 04100J7, 04100J8, 04100J9, 04100JB, 04100JC, 04100JD, 04100JF, 04100JG, 04100JH, 04100JJ, 04100JK, 04100JQ, 04100JR, 04100K6, 04100K7, 04100K8, 04100K9, 04100KB, 04100KC, 04100KD, 04100KF, 04100KG, 04100KH, 04100KJ, 04100KK, 04100KQ, 04100KR, 04100Z6, 04100Z7, 04100Z8, 04100Z9, 04100ZB, 04100ZC, 04100ZD, 04100ZF, 04100ZG, 04100ZH, 04100ZJ, 04100ZK, 04100ZQ, 04100ZR, 0410496, 0410497,0410498, 0410499, 041049B, 041049C, 041049D, 041049F, 041049G, 041049H, 041049J, 041049K, 041049Q, 041049R, 04104A6, 04104A7, 04104A8, 04104A9, 04104AB, 04104AC, 04104AD, 04104AF, 04104AG, 04104AH, 04104AJ, 04104AK, 04104AQ, 04104AR, 04104J6, 04104J7, 04104J8, 04104J9, 04104JB, 04104JC, 04104JD, 04104JF, 04104JG, 04104JH, 04104JJ, 04104JK, 04104JQ, 04104JR, 04104K6, 04104K7, 04104K8, 04104K9, 04104KB, 04104KC, 04104KD, 04104KF, 04104KG, 04104KH, 04104KJ, 04104KK, 04104KQ, 04104KR, 04104Z6, 04104Z7, 04104Z8, 04104Z9, 04104ZB, 04104ZC, 04104ZD, 04104ZF, 04104ZG, 04104ZH, 04104ZJ, 04104ZK, 04104ZQ, 04104ZR, 041C096, 041C097, 041C098, 041C099, 041C09B, 041C09C, 041C09D, 041C09F, 041C09G, 041C09H, 041C09J, 041C09K, 041C09Q, 041C0AH, 041C0AJ, 041C0AK, 041C0J6, 041C0J7, 041C0J8, 041C0J9, 041C0JB, 041C0JC, 041C0JD, 041C0JF, 041C0JG, 041C0JH, 041C0JJ, 041C0JK, 041C0JQ, 041C0K6, 041C0K7, 041C0K8, 041C0K9, 041C0KB, 041C0KC, 041C0KD, 041C0KF, 041C0KG, 041C0KH, 041C0KJ, 041C0KK, 041C0Z6, 041C0Z7,041C0Z8, 041C0Z9, 041C0ZB, 041C0ZC, 041C0ZD, 041C0ZF,041C0ZG, 041C0ZH, 041C0ZJ, 041C0ZK, 041C0ZQ, 041C496,041C497, 041C498, 041C499, 041C49B, 041C49C, 041C49D, 041C49F, 041C49G, 041C49H, 041C49J, 041C49K, 041C49Q, 041C4A6, 041C4A7, 041C4A8, 041C4A9, 041C4AB, 041C4AC, 041C4AD, 041C4AF, 041C4AG, 041C4AH, 041C4AJ, 041C4AK, 041C4AQ, 041C4JH, 041C4JJ, 041C4JK, 041C4J6, 041C4J7, 041C4J8, 041C4J9, 041C4JB, 041C4JC, 041C4JD, 041C4JF, 041C4JG, 041C4JH, 041C4JJ, 041C4JK, 041C4JQ, 041C4K6, 041C4K7, 041C4K8, 041C4K9, 041C4KB, 041C4KC, 041C4KD, 041C4KF,041C4KG, 041C4KH, 041C4KJ, 041C4KK, 041C4KQ, 041C4Z6, 041C4Z7, 041C4Z8, 041C4Z9, 041C4ZB, 041C4ZC, 041C4ZD, 041C4ZF, 041C4ZG, 041C4ZH, 041C4ZJ, 041C4ZK, 041C4ZQ, 041D098, 041D099, 041D09B, 041D09C, 041D09D, 041D09F, 041D09G, 041D09H, 041D09J, 041D09K, 041D09Q, 041D0A6, 041D0A7, 041D0A8, 041D0A9, 041D0AB, 041D0AC, 041D0AD, 041D0AF, 041D0AG, 041D0AH, 041D0AJ, 041D0AK, 041D0AQ, 041D0J6, 041D0J7, 041D0J8, 041D0J9, 041D0JB, 041D0JC, 041D0JD, 041D0JF, 041D0JG, 041D0JH, 041D0JJ, 041D0JK, 041D0JQ, 041D0K6, 041D0K7, 041D0K8, 041D0K9, 041D0KB, 041D0KC, 041D0KD, 041D0KF, 041D0KG, 041D0KH, 041D0KJ, 041D0KK, 041D0KQ, 041D0Z6, 041D0Z7, 041D0Z8, 041D0Z9, 041D0ZB, 041D0ZC, 041D0ZD, 041D0ZF, 041D0ZG, 041D0ZH, 041D0ZJ,041D0ZK, 041D0ZQ, 041D496, 041D497, 041D498, 041D499, 041D49B, 041D49C, 041D49D, 041D49F, 041D49G, 041D49H, 041D49J, 041D49K, 041D49Q, 041D4A6, 041D4A7, 041D4A8, 041D4A9, 041D4AB, 041D4AC, 041D4AD, 041D4AF, 041D4AG, 041D4AH, 041D4AJ, 041D4AK, 041D4AQ, 041D4J6, 041D4J7, 041D4J8, 041D4J9, 041D4JB, 041D4JC, 041D4JD, 041D4JF, 041D4JG, 041D4JH, 041D4JJ, 041D4JK, 041D4JQ, 041D4K7, 041D4K8, 041D4K9, 041D4KB, 041D4KC, 041D4KD, 041D4KF, 041D4KG, 041D4KH, 041D4KJ, 041D4KK, 041D4KQ, 041D4Z6, 041D4Z7, 041D4Z8, 041D4Z9, 041D4ZB, 041D4ZC, 041D4ZD, 041D4ZF, 041D4ZG, 041D4ZH, 041D4ZJ, 041D4ZK, 041D4ZQ, 041D4ZR, 041E099, 041E09B, 041E09C, 041E09D, 041E09F, 041E09G,041E09H, 041E09J, 041E09K, 041E0A9, 041E0AB, 041E0AC, 041E0AD, 041E0AF, 041E0AG, 041E0AH, 041E0AJ, 041E0AK, 041E0AP, 041E0AQ, 041E0J9, 041E0JB, 041E0JC, 041E0JD, 041E0JF, 041E0JG, 041E0JH, 041E0JJ, 041E0JK, 041E0JP, 041E0JQ, 041E0K9, 041E0KB, 041E0KC, 041E0KD, 041E0KF, 041E0KG, 041E0KH, 041E0KJ, 041E0KK, 041E0KP, 041E0KQ, 041E0Z9, 041E0ZB, 041E0ZC, 041E0ZD, 041E0ZF, 041E0ZG, 041E0ZH, 041E0ZJ, 041E0ZK, 041E0ZP, 041E0ZQ, 041E499, 041E49B, 041E49C, 041E49D, 041E49F, 041E49G, 041E49H, 041E49J, 041E49K, 041E49P, 041E49Q, 041E4A9, 041E4AB, 041E4AC, 041E4AD, 041E4AF, 041E4AG, 041E4AH, 041E4AJ, 041E4AK, 041E4AP, 041E4AQ, 041E4J9, 041E4JB, 041E4JC, 041E4JD, 041E4JF, 041E4JG, 041E4JH, 041E4JJ, 041E4JK, 041E4JP, 041E4JQ, 041E4K9, 041E4KB, 041E4KC, 041E4KD, 041E4KF, 041E4KG, 041E4KH, 041E4KJ, 041E4KK, 041E4KP, 041E4KQ, 041E4Z9, 041E4ZB, 041E4ZC, 041E4ZD, 041E4ZF, 041E4ZG, 041E4ZH, 041E4ZJ, 041E4ZK, 041E4ZP, 041E4ZQ, 041F099, 041F09B, 041F09C, 041F09D, 041F09F, 041F09G, 041F09H, 041F09J, 041F09K, 041F09P, 041F09Q, 041F0A9, 041F0AB, 041F0AC, 041F0AD, 041F0AF, 041F0AG, 041F0AH, 041F0AJ, 041F0AK, 041F0AP, 041F0AQ, 041F0J9, 041F0JB, 041F0JC, 041F0JD, 041F0JF, 041F0JG, 041F0JH, 041F0JJ, 041F0JK, 041F0JP, 041F0JQ, 041F0K9, 041F0KB, 041F0KC, 041F0KD, 041F0KF, 041F0KG, 041F0KH, 041F0KJ, 041F0KK, 041F0KP,041F0KQ, 041F0Z9, 041F0ZB, 041F0ZC, 041F0ZD, 041F0ZF, 041F0ZG, 041F0ZH, 041F0ZJ, 041F0ZK, 041F0ZP, 041F0ZQ, 041F499, 041F49B, 041F49C, 041F49D, 041F49F, 041F49G, 041F49H, 041F49J, 041F49K, 041F49P, 041F49Q, 041F4A9, 041F4AB, 041F4AC, 041F4AD, 041F4AF, 041F4AG, 041F4AH, 041F4AJ, 041F4AK, 041F4AP, 041F4AQ, 041F4J9, 041F4JB, 041F4JC, 041F4JD, 041F4JF, 041F4JG, 041F4JH, 041F4JJ, 041F4JK, 041F4JP, 041F4JQ, 041F4K9, 041F4KB, 041F4KC, 041F4KD, 041F4KF, 041F4KG, 041F4KH, 041F4KJ, 041F4KK, 041F4KP, 041F4KQ, 041F4Z9, 041F4ZB, 041F4ZC, 041F4ZD, 041F4ZF, 041F4ZG, 041F4ZH, 041F4ZJ, 041F4ZK, 041F4ZP, 041F4ZQ, 041H099, 041H09B, 041H09C, 041H09D, 041H09F, 041H09G, 041H09H, 041H09J, 041H09K, 041H09P, 041H09Q, 041H0A9, 041H0AB, 041H0AC, 041H0AD, 041H0AF, 041H0AG, 041H0AH, 041H0AJ, 041H0AK, 041H0AP, 041H0AQ, 041H0J9, 041H0JB, 041H0JC, 041H0JD, 041H0JF, 041H0JG, 041H0JH, 041H0JJ, 041H0JK, 041H0JP, 041H0JQ, 041H0K9, 041H0KB, 041H0KC, 041H0KD, 041H0KF, 041H0KG, 041H0KH, 041H0KJ, 041H0KK, 041H0KP, 041H0KQ, 041H0Z9, 041H0ZB, 041H0ZC, 041H0ZD, 041H0ZF, 041H0ZG, 041H0ZH, 041H0ZJ, 041H0ZK, 041H0ZP, 041H0ZQ, 041H499, 041H49B, 041H49C, 041H49D, 041H49F, 041H49G, 041H49H, 041H49J, 041H49K, 041H49P, 041H49Q, 041H4A9, 041H4AB, 041H4AC, 041H4AD, 041H4AF, 041H4AG, 041H4AH, 041H4AJ, 041H4AK, 041H4AP, 041H4AQ, 041H4J9, 041H4JB, 041H4JC, 041H4JD, 041H4JF, 041H4JG, 041H4JH, 041H4JJ, 041H4JK, 041H4JP, 041H4JQ, 041H4K9, 041H4KB, 041H4KC, 041H4KD, 041H4KF, 041H4KG, 041H4KH, 041H4KJ, 041H4KK, 041H4KP, 041H4KQ, 041H4Z9, 041H4ZB, 041H4ZC, 041H4ZD, 041H4ZF, 041H4ZG, 041H4ZH, 041H4ZJ, 041H4ZK, 041H4ZP, 041H4ZQ, 041J099, 041J09B, 041J09C, 041J09D, 041J09F, 041J09G, 041J09H, 041J09J, 041J09K, 041J09P,041J09Q, 041J0A9, 041J0AB, 041J0AC, 041J0AD, 041J0AF, 041J0AG, 041J0AH, 041J0AJ, 041J0AK, 041J0AP, 041J0AQ, 041J0J9, 041J0JB, 041J0JC, 041J0JD, 041J0JF, 041J0JG, 041J0JH, 041J0JJ, 041J0JK, 041J0JP, 041J0JQ, 041J0K9, 041J0KB, 041J0KC, 041J0KD, 041J0KF, 041J0KG, 041J0KH, 041J0KJ, 041J0KK, 041J0KP, 041J0KQ, 041J0Z9, 041J0ZB, 041J0ZC, 041J0ZD, 041J0ZF, 041J0ZG, 041J0ZH, 041J0ZJ, 041J0ZK, 041J0ZP, 041J0ZQ, 041J499, 041J49B, 041J49C, 041J49D, 041J49F, 041J49G, 041J49H, 041J49J, 041J49K, 041J49P, 041J49Q, 041J4A9, 041J4AB, 041J4AC, 041J4AD, 041J4AF, 041J4AG, 041J4AH, 041J4AJ, 041J4AK, 041J4AP, 041J4AQ, 041J4J9, 041J4JB, 041J4JC, 041J4JD, 041J4JF, 041J4JH, 041J4JJ, 041J4JK, 041J4JP, 041J4JQ, 041J4K9, 041J4KB, 041J4KC, 041J4KD, 041J4KF, 041J4KG, 041J4KH, 041J4KJ, 041J4KK, 041J4KP, 041J4KQ, 041J4Z9, 041J4ZB, 041J4ZC, 041J4ZD, 041J4ZF, 041J4ZG, 041J4ZH, 041J4ZJ, 041J4ZK, 041J4ZP, 041J4ZQ, 0312096, 0312097, 0312098, 0312099, 031209B, 031209C, 03120A6, 03120A7, 03120A8, 03120A9, 03120AB, 03120AC, 03120J6, 03120J7, 03120J8, 03120J9, 03120JB, 03120JC, 03120K6, 03120K7, 03120K8, 03120K9, 03120KB, 03120KC, 03120Z6, 03120Z7, 03120Z8, 03120Z9, 03120ZB, 03120ZC, 313096, 313097, 313098, 313099, 031309B, 031309C, 03130A6, 03130A7, 03130A8, 03130A9, 03130AB, 03130AC, 03130J6, 03130J7, 03130J8, 03130J9, 03130JB, 03130JC, 03130K6, 03130K7, 03130K8, 03130K9, 03130KB, 03130KC, 03130Z6, 03130Z7, 03130Z8, 03130Z9, 03130ZB, 03130ZC, 0314096, 0314097, 0314098, 0314099, 031409B, 031409C, 03140A6, 03140A7, 03140A8, 03140A9, 03140AB, 03140AC, 03140J6, 03140J7, 03140J8, 03140J9, 03140JB, 03140JC, 03140K6, 03140K7, 03140K8, 03140K9, 03140KB, 03140KC, 03140Z6, 03140Z7, 03140Z8, 03140Z9, 03140ZB, 03140ZC, 0315096, 0315097, 0315098, 0315099, 031509B, 031509C, 03150A6, 03150A7, 03150A8, 03150A9, 03150AB, 03150AC, 03150J6, 03150J7, 03150J8, 03150J9, 03150JB, 03150JC, 03150K6, 03150K7, 03150K8, 03150K9, 03150KB, 03150KC, 03150Z6, 03150Z7, 03150Z8, 03150Z9, 03150ZB, 03150ZC, 0316096, 0316097, 0316098, 0316099, 031609B, 031609C, 03160A6, 03160A7, 03160A8, 03160A9, 03160AB, 03160AC, 03160J6, 03160J7, 03160J8, 03160J9, 03160JB, 03160JC, 03160K6, 03160K7, 03160K8, 03160K9, 03160KB, 03160KC, 03160Z6, 03160Z7, 03160Z8, 03160Z9, 03160ZB, 03160ZC, 041K09H, 041K09J, 041K09K, 041K09L, 041K09M, 041K09N, 041K09P, 041K09Q, 041K0AH, 041K0AJ, 041K0AK, 041K0AL, 041K0AM, 041K0AN, 041K0AP, 041K0AQ, 041K0JH, 041K0JJ, 041K0JK, 041K0JL, 041K0JM, 041K0JN, 041K0JP, 041K0JQ, 041K0KH, 041K0KJ, 041K0KK, 041K0KL, 041K0KM, 041K0KN, 041K0KP, 041K0KQ, 041K0ZH, 041K0ZJ, 041K0ZK, 041K0ZL, 041K0ZM, 041K0ZN, 041K0ZP, 041K0ZQ, 041K49H, 041K49J, 041K49K, 041K49L, 041K49M, 041K49N, 041K49P, 041K49Q, 041K4AH, 041K4AJ, 041K4AK, 041K4AL, 041K4AM, 041K4AN, 041K4AP, 041K4AQ, 041K4JH, 041K4JJ, 041K4JK, 041K4JL, 041K4JM, 041K4JN, 041K4JP, 041K4JQ, 041K4KH, 041K4KJ, 041K4KK, 041K4KL, 041K4KM, 041K4KN, 041K4KP, 041K4KQ, 041K4ZH, 041K4ZJ, 041K4ZK, 041K4ZL, 041K4ZM, 041K4ZN, 041K4ZP, 041K4ZQ, 041L09H, 041L09J, 041L09K, 041L09L, 041L09M, 041L09N, 041L09P, 041L09Q, 041L0AH, 041L0AJ, 041L0AK, 041L0AL, 041L0AM, 041L0AN, 041L0AP, 041L0AQ, 041L0JH, 041L0JJ, 041L0JK, 041L0JL, 041L0JM, 041L0JN, 041L0JP, 041L0JQ, 041L0KH, 041L0KJ, 041L0KK, 041L0KL, 041L0KM, 041L0KN, 041L0KP, 041L0KQ, 041L0ZH, 041L0ZJ, 041L0ZK, 041L0ZL, 041L0ZM, 041L0ZN, 041L0ZP, 041L0ZQ, 041L49H, 041L49J, 041L49K, 041L49L, 041L49M, 041L49N, 041L49P, 041L49Q, 041L4AH, 041L4AJ, 041L4AK, 041L4AL, 041L4AM, 041L4AN, 041L4AP, 041L4AQ, 041L4JH, 041L4JJ, 041L4JK, 041L4JL, 041L4JM, 041L4JN, 041L4JP, 041L4JQ, 041L4KH, 041L4KJ, 041L4KK, 041L4KL, 041L4KM, 041L4KN, 041L4KP, 041L4KQ, 041L4ZH, 041L4ZJ, 041L4ZK, 041L4ZL, 041L4ZM, 041L4ZN, 041L4ZP, 041L4ZQ, 041M09L, 041M09M, 041M09P, 041M09Q, 041M0AL, 041M0AM, 041M0AP, 041M0AQ, 041M0JL, 041M0JM, 041M0JP, 041M0JQ, 041M0KL, 041M0KM, 041M0KP, 041M0KQ, 041M0ZL, 041M0ZM, 041M0ZP, 041M0ZQ, 041M49L, 041M49M, 041M49P, 041M49Q, 041M4AL, 041M4AM, 041M4AP, 041M4AQ, 041M4JL, 041M4JM, 041M4JP, 041M4JQ, 041M4KL, 041M4KM, 041M4KP, 041M4KQ, 041M4ZL, 041M4ZM, 041M4ZP, 041M4ZQ, 041N09L, 041N09M, 041N09P, 041N09Q, 041N0AL,041N0AM, 041N0AP, 041N0AQ, 041N0JL, 041N0JM, 041N0JP, 041N0JQ, 041N0KL, 041N0KM, 041N0KP, 041N0KQ, 041N0ZL, 041N0ZM, 041N0ZP, 041N0ZQ, 041N49L, 041N49M, 041N49P, 041N49Q, 041N4AL, 041N4AM, 041N4AP, 041N4AQ, 041N4JL, 041N4JM, 041N4JP, 041N4JQ, 041N4KL, 041N4KM, 041N4KP, 041N4KQ, 041N4ZL, 041N4ZM, 041N4ZP, 041N4ZQ, 041T09P, 041T09Q, 041T0AP, 041T0AQ, 041T0JP, 041T0JQ, 041T0KP, 041T0KQ, 041T0ZP, 041T0ZQ, 041T49P, 041T49Q, 041T4AP, 041T4AQ, 041T4JP, 041T4JQ, 041T4KP, 041T4KQ, 041T4ZP, 041T4ZQ, 041U09P, 041U09Q, 041U0AP, 041U0AQ, 041U0JP, 041U0JQ, 041U0KP, 041U0KQ, 041U0ZP, 041U0ZQ, 041U49P, 041U49Q, 041U4AP, 041U4AQ, 041U4JP, 041U4JQ, 041U4KP, 041U4KQ, 041U4ZP, 041U4ZQ, 041V09P, 041V09Q, 041V0AP, 041V0AQ, 041V0JP, 041V0JQ, 041V0KP, 041V0KQ, 041V0ZP, 041V0ZQ, 041V49P, 041V49Q, 041V4AP, 041V4AQ, 041V4JP, 041V4JQ, 041V4KP, 041V4KQ, 041V4ZP, 041V4ZQ, 041W09P, 041W09Q, 041W0AP, 041W0AQ, 041W0JP, 041W0JQ, 041W0KP, 041W0KQ, 041W0ZP, 041W0ZQ, 041W49P, 041W49Q, 041W4AP, 041W4AQ, 041W4JP, 041W4JQ, 041W4KP, 041W4KQ, 041W4ZP, 041W4ZQ, 047C041, 047C04Z, 047C0D1, 047C0DZ, 047C0Z1, 047C0ZZ, 047C341, 047C34Z, 047C3D1, 047C3DZ, 047C3Z1, 047C3ZZ, 047C441, 047C44Z, 047C4D1, 047C4DZ, 047C4Z1, 047C4ZZ, 047D041, 047D04Z, 047D0D1, 047D0DZ, 047D0Z1, 047D0ZZ, 047D341, 047D34Z, 047D3D1, 047D3DZ, 047D3Z1, 047D3ZZ, 047D441, 047D44Z, 047D4D1, 047D4DZ, 047D4Z1, 047D4ZZ, 047E041, 047E04Z, 047E0D1, 047E0DZ, 047E0Z1, 047E0ZZ, 047E341, 047E34Z, 047E3D1, 047E3DZ, 047E3Z1, 047E3ZZ, 047E441, 047E44Z, 047E4D1, 047E4DZ, 047E4Z1, 047E4ZZ, 047F041, 047F04Z, 047F0D1, 047F0DZ, 047F0Z1, 047F0ZZ, 047F341, 047F34Z, 047F3D1, 047F3DZ, 047F3Z1, 047F3ZZ, 047F441, 047F44Z, 047F4D1, 047F4DZ, 047F4Z1, 047F4ZZ, 047H041, 047H04Z, 047H0D1, 047H0DZ, 047H0Z1, 047H0ZZ, 047H341, 047H34Z, 047H3D1, 047H3DZ, 047H3Z1, 047H3ZZ, 047H441, 047H44Z, 047H4D1, 047H4DZ, 047H4Z1, 047H4ZZ, 047J041, 047J04Z, 047J0D1, 047J0DZ, 047J0Z1, 047J0ZZ, 047J341, 047J34Z, 047J3D1, 047J3DZ, 047J3Z1, 047J3ZZ, 047J441, 047J44Z, 047J4D1, 047J4DZ, 047J4Z1, 047J4ZZ, 047K04Z, 047K0DZ, 047K0ZZ, 047K34Z, 047K3DZ, 047K3ZZ, 047K44Z, 047K4DZ, 047K4ZZ, 047L04Z, 047L0DZ, 047L0ZZ, 047L34Z, 047L3DZ, 047L3ZZ, 047L44Z, 047L4DZ, 047L4ZZ, 047M04Z, 047M0DZ, 047M0ZZ, 047M34Z, 047M3DZ, 047M3ZZ, 047M44Z, 047M4DZ, 047M4ZZ, 047N04Z, 047N0DZ, 047N0ZZ, 047N34Z, 047N3DZ, 047N3ZZ, 047N44Z, 047N4DZ, 047N4ZZ, 047P041, 047P04Z, 047P0D1, 047P0DZ, 047P0Z1, 047P0ZZ, 047P341, 047P34Z, 047P3D1, 047P3DZ, 047P3Z1, 047P3ZZ, 047P441, 047P44Z, 047P4D1, 047P4DZ, 047P4Z1, 047P4ZZ, 047Q041, 047Q04Z, 047Q0D1, 047Q0DZ, 047Q0Z1, 047Q0ZZ, 047Q341, 047Q34Z, 047Q3D1, 047Q3DZ, 047Q3Z1, 047Q3ZZ, 047Q441, 047Q44Z, 047Q4D1, 047Q4DZ, 047Q4Z1, 047Q4ZZ, 047R041, 047R04Z, 047R0D1,047R0DZ, 047R0Z1, 047R0ZZ, 047R341, 047R34Z, 047R3D1, 047R3DZ, 047R3Z1, 047R3ZZ, 047R441, 047R44Z, 047R4D1,047R4DZ, 047R4Z1, 047R4ZZ, 047S041, 047S04Z, 047S0D1, 047S0DZ, 047S0Z1, 047S0ZZ, 047S341, 047S34Z, 047S3D1,047S3DZ, 047S3Z1, 047S3ZZ, 047S441, 047S44Z, 047S4D1,047S4DZ, 047S4Z1, 047S4ZZ, 047T041, 047T04Z, 047T0D1,047T0DZ, 047T0Z1,047T0ZZ, 047T341, 047T34Z, 047T3D1, 047T3DZ, 047T3Z1, 047T3ZZ, 047T441, 047T44Z, 047T4D1,047T4DZ, 047T4Z1, 047T4ZZ, 047U041, 047U04Z, 047U0D1, 047U0DZ, 047U0Z1, 047U0ZZ, 047U341, 047U34Z, 047U3D1,047U3DZ, 047U3Z1, 047U3ZZ, 047U441, 047U44Z, 047U4D1,047U4DZ, 047U4Z1, 047U4ZZ, 047V041, 047V04Z, 047V0D1,047V0DZ, 047V0Z1, 047V0ZZ, 047V341, 047V34Z, 047V3D1,047V3DZ, 047V3Z1, 047V3ZZ, 047V441, 047V44Z, 047V4D1,047V4DZ, 047V4Z1, 047V4ZZ, 047W041, 047W04Z, 047W0D1,047W0DZ, 047W0Z1, 047W0ZZ, 047W341, 047W34Z, 047W3D1,047W3DZ, 047W3Z1, 047W3ZZ, 047W441, 047W44Z, 047W4D1, 047W4DZ, 047W4Z1, 047W4ZZ, 047Y041, 047Y04Z, 047Y0D1,047Y0DZ, 047Y0Z1, 047Y0ZZ, 047Y341, 047Y34Z, 047Y3D1, 047Y3DZ, 047Y3Z1, 047Y3ZZ, 047Y441, 047Y44Z, 047Y4D1, 047Y4DZ, 047Y4Z1, 047Y4ZZ  **ICD-9-CM**: 444.0, 444.01, 444.09, 444.22, 444.81, 99.10, 84.13, 84.14, 84.15, 84.16, 84.17, 38.08, 38.16, 38.18, 38.38, 38.48, 38.68, 38.88, 39.25, 39.29, 39.50, 440.0x, 440.1, 440.2x, 440.29, 440.3x, 440.30, 440.31, 440.32, 440.4x, 440.8x, 440.9x, 445.01, 445.02, 445.81, 445.89, 362.30, 440.0, 440.20, 440.30, 443.9, 441, 441.0, 441.00, 441.01, 441.02, 441.03, 441.2, 441.4, 441.7, 441.9, 442, 442.0, 442.1, 442.2, 442.3, 442.8, 442.81, 442.82, 442.83, 442.84, 442.89, 442.9, 443, 443.1, 443.2, 443.21, 443.22, 443.23, 443.24, 443.29, 443.8, 443.81, 443.89, 445.xx, 445.0, 445.01, 445.02, 445.8, 445.81, 445.89  **CPT**: 34201, 34203, 37184, 37211, 37213, 27590, 27591, 27592, 27598, 27880, 27881, 27882, 27886, 27888, 27889, 35302, 35303, 35304, 35305, 35351, 35355, 35361, 35363, 35371, 35372, 35381, 35480, 35481, 35482, 35483, 35485, 35521, 35537, 35538, 35539, 35540, 35541, 35546, 35548, 35549, 35551, 35556, 35558, 35563, 35565, 35566, 35570, 35571, 35583, 35585, 35587, 35621, 35623, 35641, 35646, 35647, 35651, 35654, 35656, 35661, 35663, 35665, 35666, 35671, 35875, 35876, 35454, 35456, 35459, 35470, 35473, 35474, 35491, 35492, 35493, 35495, 37205, 37206, 37207, 37208, 37220, 37221, 37224, 37225, 37226, 37227, 37228, 37229, 37230, 37231 |
| Other ASCVD | **ICD-10-CM:** I25.6, I25.10, I25.5, I25.810, I25.811, I25.812, I25.82, I25.83, I25.89, I25.84, I25.9  **ICD-9:** 414, 414.01, 414.02, 414.03, 414.04, 414.05, 414.06, 414.07, 414.1, 414.12, 414.19, 414.2, 414.3, 414.4, 414.8, 414.9, 429.79 |

ASCVD, atherosclerotic cardiovascular disease; CM, clinical modification; CPT, Current Procedural Terminology; HCPCS, Healthcare Common Procedure Coding System; ICD, International Classification of Diseases; PCS, Procedure Coding System

Supplementary Table 2. Definition of major ASCVD events and high-risk conditions in the 2018 ACC/AHA blood cholesterol guideline

| Definitions for major ASCVD events | |
| --- | --- |
| ACC/AHA criteria | Operational definition |
| 1) Recent ACS (within the past 12 months) | Defined as an event of MI (IP) or unstable angina hospitalization (IP). The other required criteria (a second major ASCVD event or 2 high-risk conditions) must occur before or within 12 months following the recent ACS event. |
| 2) History of MI (other than recent ACS event listed above) | Patients with at least 1 claim with a history of MI, assessed by MI event codes or status codes (exclude recent ACS event in criteria 1). |
| 3) History of IS | Patients with at least 1 event of IS assessed through IS events or IS status codes. |
| 4) Symptomatic PAD (history of claudication with ABI <0.85, or previous revascularization or amputation) | Patients with a diagnosis of symptomatic PAD event. |
| Definitions for risk factors | |
| ACC/AHA criteria | Operational definition |
| 1) Age ≥65 years | Age at which the patient is ≥65 years during the index period. |
| 2) Heterozygous familial hypercholesterolemia (HeFH) | 1. Patients with 1 LDL-C ≥190 mg/dL AND a second LDL-C ≥190 mg/dL or an on-treatment LDL-C ≥130 mg/dL, OR  2. At least 1 claim with a diagnosis code E78.01. |
| 3) History of prior CABG or PCI outside of major ASCVD event(s) | a. In patients with no prior MI/IS: Any revascularization event occurring during the study period.  b. In patients with prior MI/IS: Revascularization occurring during the 30-day period after an MI will not be considered. Patients with at least 1 revascularization procedure occurring outside the 30-day period after MI/IS during the study period. |
| 4) Diabetes mellitus (T2DM) | Assessed by using diagnosis codes or anti-diabetic therapy during the 5-year pre-index period. |
| 5) Hypertension | Assessed by using diagnosis codes during the 5-year pre-index period. |
| 6) CKD (eGFR 15-59 mL/min/1.73 m2) | CKD stage III-IV assessed using ICD claims during the study period (ICD-9 diagnosis codes for CKD, stage III-IV: 585.3, 585.4; ICD-10 diagnosis codes: N18.3, N18.4). |
| 7) Current smoking | Patients with a diagnosis for smoking or a history of smoking during the study period. |
| 8) Persistently elevated LDL-C (LDL-C ≥100) despite maximally tolerated statin therapy and ezetimibe | Defined as patients with 2 sequential LDL-C values over 100 mg/dL, evidence of any-intensity statin or ezetimibe within 1 year of most recent LDL-C 100+ mg/dL. |
| 9) History of CHF | Defined as patients with at least 1 claim for CHF during the study period (ICD-9 diagnosis codes for heart failure: 428.x or 428.xx; ICD-10 diagnosis codes: I50.x, I50.xx). |

ABI, ankle-brachial index; ACC, American College of Cardiology; ACS, acute coronary syndrome; AHA, American Heart Association; ASCVD, atherosclerotic cardiovascular disease; CABG, coronary artery bypass graft; CHF, congestive heart failure; CKD, chronic kidney disease; eGFR, estimated glomerular filtration rate; ICD, International Classification of Diseases; IP, inpatient; IS, ischemic stroke; LDL-C, low-density lipoprotein cholesterol; MI, myocardial infarction; PAD, peripheral arterial disease; PCI, percutaneous coronary intervention; T2DM, type 2 diabetes mellitus

Supplementary Table 3. Baseline demographics and clinical characteristics in VHR and non-VHR ASCVD cohorts before IDS matching

|  | VHR ASCVD (N = 117,460) | Non-VHR ASCVD (N = 306,172) |
| --- | --- | --- |
| Demographics | | |
| Mean (SD) age, years | 63.1 (10.2) | 62.1 (10.7) |
| Men, % | 61.1 | 55.7 |
| Geographic region, % | | |
| Northeast | 17.2 | 17.3 |
| Midwest | 11.5 | 10.6 |
| South | 64.4 | 66.2 |
| West | 6.9 | 5.9 |
| Payer type, % | | |
| Commercial | 79.3 | 80.6 |
| Medicare | 16.7 | 15.6 |
| Other | 4.1 | 3.9 |
| Clinical characteristics | | |
| Mean (SD) LDL-C, mg/dL | 110.5 (38.9) | 107.2 (36.9) |
| Mean (SD) LDL-C, mmol/L | 2.9 (1.0) | 2.8 (1.0) |
| Distribution of LDL-C, % | | |
| LDL-C <70 mg/dL (<1.8 mmol/L) | 13.4 | 14.3 |
| LDL-C 70-99 mg/dL (1.8-2.6 mmol/L) | 29.3 | 31.6 |
| LDL-C 100-129 mg/dL (2.6-3.3 mmol/L) | 29.2 | 29.3 |
| LDL-C 130-159 mg/dL (3.4-4.1 mmol/L) | 17.6 | 16.3 |
| LDL-C 160-189 mg/dL (4.1-4.9 mmol/L) | 7.1 | 6.0 |
| LDL-C >189 mg/dL (>4.9 mmol/L) | 3.5 | 2.5 |
| ASCVD type, % | | |
| MI | 34.0 | 6.5 |
| UA hospitalization | 1.7 | 0.4 |
| Stable angina hospitalization | 4.0 | 3.8 |
| IS | 26.8 | 8.8 |
| TIA | 9.8 | 11.0 |
| PCI | 16.0 | 7.6 |
| CABG | 5.6 | 4.3 |
| PAD | 13.9 | 29.0 |
| Other ASCVD | 44.9 | 53.4 |
| Current LLT use, % | | |
| Any statin and/or ezetimibe | 43.8 | 44.7 |
| Statin only | 40.8 | 41.8 |
| High-intensity statin | 11.4 | 11.3 |
| Medium-intensity statin | 25.2 | 26.0 |
| Low-intensity statin | 4.3 | 4.4 |
| Statin + ezetimibe | 2.4 | 2.3 |
| High-intensity statin | 1.0 | 1.0 |
| Medium-intensity statin | 1.3 | 1.3 |
| Low-intensity statin | 0.1 | 0.1 |
| Ezetimibe only | 0.6 | 0.6 |

ASCVD, atherosclerotic cardiovascular disease; CABG, coronary artery bypass graft; IDS, incidence density sampling; IS, ischemic stroke; LDL-C, low-density lipoprotein cholesterol; LLT, lipid-lowering therapy; MI, myocardial infarction; PAD, peripheral arterial disease; PCI, percutaneous coronary intervention; SD, standard deviation; TIA, transient ischemic attack; UA, unstable angina; VHR, very high-risk
